# Prevalence of self-reported hearing difficulty on the Revised Hearing Handicap Inventory and associated factors

**DOI:** 10.1186/s12877-024-04901-w

**Published:** 2024-06-12

**Authors:** Lauren K. Dillard, Lois J. Matthews, Judy R. Dubno

**Affiliations:** Department of Otolaryngology-Head & Neck Surgery, Medical University of South Carolina, 135 Rutledge Avenue, MSC 550, Charleston, SC 29425 USA

**Keywords:** Hearing loss, Patient reported outcome measures, Demographics, Cohort study, Self-report, Audiology

## Abstract

**Background:**

Hearing loss is common in aging adults and is an important public health concern. Self-reported measures of hearing difficulty are often used in research and clinical practice, as they capture the functional impacts of hearing loss on individuals. However, little research has evaluated the prevalence or factors associated with self-reported hearing difficulty. Therefore, the purpose of this study was to determine the prevalence of self-reported hearing difficulty, measured by the Revised Hearing Handicap Inventory (RHHI), and associated factors.

**Methods:**

This study was conducted in a community-based cohort study based in Charleston, SC. We determined the prevalence of RHHI self-reported hearing difficulty (score ≥ 6 points) and evaluated associated factors with logistic regression models. Results are presented as odds ratios (OR) with corresponding 95% confidence intervals (95% CI).

**Results:**

There were 1558 participants included in this study (mean age 63.7 [SD 14.4], 56.9% female, 20.0% Minority race). The prevalence of RHHI self-reported hearing difficulty was 48.8%. In a multivariable model, older age (per + 1 year; OR 0.97 [95% CI 0.96, 0.98]), Minority (vs. White) race (OR 0.68 [95% CI 0.49, 0.94]), and speech-in-noise scores that are better than predicted (OR 0.99 [95% CI 0.98, 1.00]) were associated with lower odds of RHHI self-reported hearing difficulty. Furthermore, female (vs. male) sex (OR 1.39 [95% CI 1.03, 1.86]), higher PTA in the worse ear (per + 1 dB; OR 1.10 [95% CI 1.09, 1.12]), more comorbid conditions (vs. 0; 1 condition: OR 1.50 [95% CI 1.07, 2.11]; 2 conditions: OR 1.96 [95% CI 1.32, 2.93]; 3 + conditions: OR 3.00 [95% CI 1.60, 5.62]), noise exposure (OR 1.54 [95% CI 1.16, 2.03]), bothersome tinnitus (OR 2.16 [95% CI 1.59, 2.93]), and more depressive symptoms (OR 1.04 [95% CI 1.01, 1.07]) were associated with higher odds of RHHI self-reported hearing difficulty.

**Conclusions:**

The prevalence of RHHI self-reported hearing difficulty is high, and associated factors included demographics, audiometric hearing and other hearing-related factors, and physical and mental health. The RHHI likely captures functional impacts of hearing loss that are not captured by audiometry alone. Study findings can support the correct interpretation of the RHHI in research and clinical settings.

**Supplementary Information:**

The online version contains supplementary material available at 10.1186/s12877-024-04901-w.

## Background

Age-related hearing loss (ARHL) is common in older adults and impactful to health and well-being [1–4]. In clinical and research settings, ARHL is often defined by pure-tone audiometry, although audiometry alone does not capture functional impacts of hearing loss on individuals and therefore should be supplemented with self-reported measures of hearing [5–7]. The World Health Organization defines disability and health as capturing impairment of body functions and structures (physical impairment), but also the impacts of disability on function, activity limitations, and participation restrictions [8]. Audiometric hearing loss is considered a measure of impairment, whereas self-reported hearing difficulty is a multidimensional construct capturing function, activity limitations, and participation restrictions, and therefore the burden of hearing loss, which cannot be determined from audiometry alone [6]. 

Measures of self-reported hearing difficulty are important for clinical practice and research, including epidemiological studies. They quantify impacts of hearing loss and serve as patient-reported outcome measures that, for example, assess success of an intervention [7, 9]. The Revised Hearing Handicap Inventory (RHHI) is a psychometrically robust measure of hearing difficulty adapted from the widely used Hearing Handicap Inventory for the Elderly (HHIE) and Adults (HHIA) [HHIE/A] [10–13]. The HHIE/A has been measured in several epidemiological cohort studies, and is used to promote clinical decision making, indicating the RHHI can be used similarly [14, 15]. RHHI self-reported hearing difficulty is related to audiometric hearing loss, yet as discussed above, likely captures more complex constructs of hearing impairment beyond what is defined by audiometry alone [13]. 

Several epidemiological studies have evaluated factors associated with audiometric hearing or subjective hearing evaluated with single questions [14–17]. However, some of these studies do not have audiometric hearing data; therefore, it is not possible to distinguish whether associations capture relationships between given factors and audiometric hearing (not measured) and/or self-reported hearing difficulty [16, 17]. Few studies have reported the prevalence of self-reported hearing handicap or difficulty, determined by standardized measures such as the HHIE/A or RHHI, or the factors associated with self-reported hearing handicap or difficulty [18, 19]. One study conducted in adults aged 65 or older found that audiometric hearing, marital status, and self-reported health were associated with hearing handicap on the HHIE screening version [18]. A study conducted in adults aged 18–74 years in the Hispanic Community Health Study/Study of Latinos showed that audiometric hearing, acculturation, sex, and income were associated with hearing handicap on the screening versions of the HHIE and HHIA [19]. 

Given that there are few studies that report factors associated with self-reported hearing difficulty, as defined by standardized measures, additional research in samples of the general population is needed to understand these relationships. Therefore, the purpose of this study was to determine the prevalence of RHHI self-reported hearing difficulty and associated factors in a community-based cohort study.

## Methods

### Study population

The Medical University of South Carolina (MUSC) Longitudinal Cohort Study of ARHL is an ongoing (1988-current) community-based cohort study based in Charleston, SC. Previous publications have described the cohort and methods in detail [20–23]. Briefly, participants must be aged 18 years or older, and in good general health with no evidence of conductive hearing loss or active otologic or neurologic disease.

This cross-sectional study uses baseline data. Participants are enrolled continuously, and the baseline examination consists of three to six laboratory visits, each of which lasts approximately 1.5 h. The baseline examination includes comprehensive measures of hearing, and health and hearing-related history. The battery of tests includes pure-tone air conduction audiometry, speech recognition measures, and additional auditory tests such as otoacoustic emissions and auditory brainstem responses. The battery of tests also includes surveys including the HHIE/A, the Patient-Reported Outcomes Measures Information System (PROMIS, Item Bank v1.0) [10, 11, 24], and other questionnaires capturing demographics and self and family hearing and medical histories [20–23]. Participants attend follow-up examinations every 2 to 3 years after baseline, during which nearly all measures are repeated.

There are currently 1,776 participants with baseline data. To be included in this study, participants must have complete data on the RHHI (described below). All participants provided written informed consent and all protocols for this study were approved by the Institutional Review Board at MUSC.

### Audiometric testing and hearing-related measures

Pure-tone audiometric thresholds at frequencies 0.25, 0.5, 1.0, 2.0, 3.0, 4.0, 6.0, and 8.0 kHz were measured with a clinical audiometer equipped with TDH-39 headphones (Telephonics Corporation, Farmingdale, NY, USA) in a sound-treated booth. All audiological equipment is calibrated annually to the appropriate American National Standards Institute (ANSI) standards by manufacturers’ representatives [25]. 

Thresholds were measured in 5-dB steps following American Speech-Language-Hearing Association standards [26]. A pure-tone average (PTA) was calculated from thresholds at 0.5, 1.0, 2.0, and 4.0 kHz in each ear. PTA in the worse ear was used as a continuous variable in analyses. For descriptive purposes, worse ear PTA was categorized as mild (> 25–40 dB HL), moderate (> 40–55 dB HL), moderately severe (> 55–70 dB HL) and severe or profound (> 70 dB HL). Audiometric hearing loss was defined as PTA > 25 dB HL in the worse ear. PTA in the worse ear captures asymmetrical and unilateral hearing losses, in addition to bilateral hearing acuity. A small proportion of participants in this cohort had asymmetrical (3.5%) or unilateral (0.4%) hearing loss, defined as a difference in PTA ≥ 15 dB between ears when audiometric hearing loss was present or absent, respectively. PTA in the worse ear is a definition used in several epidemiological studies of the general population [12–14]. Speech-reception thresholds (SRT) were measured with recorded 2-syllable words, with lower SRTs indicating better hearing for speech [27]. The presentation level for the Staggered Spondaic Word test (SSW), a dichotic listening task, was 50 dB above SRT or (if needed) comfort level (but not < 30 dB above the SRT or > 90 dB HL) [28]. SSW scores are presented as the percent total error (uncorrected for word recognition) and higher scores indicate worse performance.

Participants underwent the Speech Perception in Noise (SPIN) test [29]. Fifty-item lists of intermingled high- and low-context sentences were presented in each ear 50 dB above the estimated babble threshold at a + 8 dB signal-to-babble ratio [30]. Individual participants’ babble thresholds were not measured; rather, babble thresholds were calculated (estimated) based on individual participants’ pure-tone thresholds at frequencies 0.5, 1.0, 2.0, and 4.0 kHz, as described by Bilger et al. [31]. To assess scores while controlling for reduced audibility, SPIN scores are compared to scores predicted by the articulation index (AI) speech-audibility metric [20, 32, 33]. Because no correction factors (such as distortion factor) were used in calculating the AI, the AI is equivalent to the Speech Intelligibility Index [33]. SPIN scores from low-context sentences only are presented as the observed *minus* predicted values in the worse ear and are henceforth referred to as speech-in-noise scores. A positive difference indicates that observed scores are better than predicted and a negative difference indicates scores are poorer than predicted.

Noise exposure is defined as a positive history of occupational (including military) noise exposure and/or regular firearm use for at least one year, and/or acute acoustic trauma from impulse noise [22]. Participants were asked if they experienced tinnitus. If they responded yes, they were asked how many days per month it bothered them (range 0 to 30 days). Bothersome tinnitus was defined as tinnitus considered to be bothersome at least one day per month. Notably, definitions of tinnitus vary widely across studies, although the definition used in this study produced a similar prevalence estimate to other epidemiological studies [34].

### Outcome: RHHI self-reported hearing difficulty

The HHIE/A was administered to individuals aged ≥ 60 and < 60 years, respectively. The HHIE/A each consist of 25 questions (3 questions differ between tools) and possible responses include yes, sometimes, or no, which are assigned scores of 4, 2, and 0, respectively [10, 11]. The scores are summed (range 0-100) and higher scores indicate greater perceived hearing difficulties.

The RHHI was created from the 22 questions common to both the HHIE and HHIA via psychometric analyses [12]. The RHHI is an 18-item unidimensional scale of self-perceived hearing difficulty, and the RHHI-S is the corresponding 10-item screening tool that consists of a subset of questions on the RHHI. The responses and scoring methods for the RHHI and RHHI-S are the same as described above, and scores range from 0 to 72 or 40, respectively. Details on the administration and development of these questionnaires have been published [12, 13]. RHHI and RHHI-S scores were derived from HHIE/A responses. RHHI and RHHI-S self-reported hearing difficulty were defined as scores ≥ 6 [13]. 

### Demographic factors

Participants reported their age, sex assigned at birth (male/female), race (according to US Census Bureau classifications) [35], education, and occupation. Race was categorized as White or racial Minority to ensure appropriate statistical power for analyses. Self-reported marital status was categorized as married, divorced or separated, single, or widowed.

A proxy for socioeconomic position (SEP) was determined from participants’ number of years of education and occupation at the baseline examination, and is described in detail elsewhere [13, 23, 36]. Briefly, SEP classifications were determined for education and occupation, separately, by classifying education and occupation as low, mid, or high, then these classifications were combined to form a single SEP proxy. Educational classifications were low (high school degree or less; ≤12 years), mid (associate degree or some college; 12 to < 16 years) or high (college graduate or more; 16 + years). Occupational classifications were derived from US annual salary data, which were used to classify occupations as low (operatives; labors and helpers; service workers), mid (technicians; sales workers; administrative support workers; craft workers), and high (executive/senior officials and managers; first/mid-level officials and managers; professionals) [23]. 

### Physical and mental health

Health data were self-reported. Participants reported history of (current or past) the following comorbid conditions, thyroid disease, kidney problems, cancer or tumor (any type), and arthritis [23], and the number of comorbid conditions was categorized as 0, 1, 2, or 3+. Participants reported history of diabetes (type 1 or 2). The presence of cardiovascular conditions was defined as history of stroke, chest pain, heart disease, or high blood pressure. Smoking was categorized as never, current, or past. Body mass index (BMI) was calculated as self-reported weight in kilograms (kg) divided by height in meters (m) squared.

The PROMIS was administered via paper short forms [24]. We used the total scores for the Emotional Distress-Depression (range 8–40 points; higher score indicates more depressive symptoms) and Satisfaction with Participation in Discretionary Social Activities (range 7–35 points; higher score indicates more satisfaction in social activities) subscales.

### Statistical methods

All statistical analyses were conducted in SAS version 9.4 software (Cary, NC). Hot-deck imputation, using the simple random samples with replacement method, was used to avoid losing cases affected by missing covariate data (i.e., the factors described above; outcome data [RHHI] were not imputed) [37]. In this method, observed values from the sample (donors) were used to impute missing values (recipients). Donor units were randomly selected based on their similarity to recipient units in terms of hearing, demographics, and health history [38]. 

We used chi-square for categorical variables and one-way analysis of variance (for continuous variables) to determine (i) demographic differences (age, sex, race, SEP proxy) for participants included in this study and excluded from this study (based on inclusion criteria defined above), and (ii) differences in sample characteristics between participants with and without RHHI self-reported hearing difficulty. Results are presented as *p*-values. For descriptive purposes, we plot relationships of baseline age and baseline PTA for participants with and without RHHI self-reported hearing difficulty, separately, and together. For these relationships, we present linear regression coefficients with corresponding 95% confidence intervals (CI). We also present differences in the regression coefficients for participants with and without RHHI self-reported hearing difficulty, which were determined by the significance of interaction terms of PTA and RHHI self-reported hearing difficulty in those models.

Logistic regression models were used to evaluate factors associated with RHHI self-reported hearing difficulty. First, base models were adjusted for age, sex, and PTA. Second, factors significant at *p* < 0.10 in base models were included in a multivariable model. At this step, a more liberal cutoff of *p* < 0.10 (rather than *p* < 0.05) was chosen as our goal was to identify possible predictor variables rather than to test a pre-specified hypothesis [17, 39]. Results are presented as odds ratios (OR) with corresponding 95% CI. We identified factors hypothesized to be associated with RHHI self-reported hearing difficulty from existing literature [1, 7, 13, 14, 17]. For all analyses, except the modeling procedure described above, statistical significance is defined by *p* < 0.05.

### Supplementary and sensitivity analyses

We repeated the modeling procedure described above in females and males, separately, and White and Minority participants, separately, to determine if there were sex or race differences in factors associated with RHHI self-reported hearing difficulty. We hypothesized there may be sex or race differences in factors associated with RHHI hearing difficulty, in part, given evidence that sex and race modify the agreement between self-reported hearing difficulty and audiometric hearing [40]. No studies (to the authors’ knowledge) have evaluated sex or race differences in factors associated with self-reported hearing difficulty, independent of audiometric hearing. To determine if there were differences in factors associated with the screening tool (RHHI-S), we repeated the modeling procedure described above using RHHI-S self-reported hearing difficulty (score ≥ 6) as the outcome [12]. 

## Results

Of the 1776 participants with baseline data, 1558 had complete RHHI data and were included in this study. As compared to participants with complete RHHI data (included in this study), participants with missing RHHI data (excluded from this study; *n* = 218, 12.3%) were more likely to be younger (*p* < 0.01) and have a lower (better) PTA (*p* < 0.01), but did not differ by sex, race, or SEP proxy (*p* > 0.05).

Table 1 shows characteristics of the 1558 participants included in this study, overall and by RHHI self-reported hearing difficulty. Participants’ mean age was 63.7 (SD 14.4) years, 56.9% were female and 20.0% were Minority race (18.9% of the sample were Black or African American). As compared to those without RHHI self-reported hearing difficulty, participants with RHHI self-reported hearing difficulty were more likely to be older, male, White, married, and have a higher (worse) PTA. Additionally, they were more likely to have more comorbid conditions, history of noise exposure, bothersome tinnitus, cardiovascular conditions, speech-in-noise scores that are poorer than predicted and poorer SSW scores, more depressive symptoms, and less satisfaction in social activities.

**Table 1:** Study sample characteristics (*n* = 1558)

	Entire Sample (*n* = 1558)	No RHHI self-reported hearing difficulty (*n* = 798)	RHHI self-reported hearing difficulty (*n* = 760)	***p***-value
Characteristic	Mean (SD) or n (%)	Mean (SD) or n (%)	Mean (SD) or n (%)	
Age (years)	63.7 (14.4)	60.8 (15.4)	66.7 (12.0)	< 0.01
Sex				< 0.01
Female	887 (56.9%)	498 (62.4%)	389 (51.2%)	
Male	671 (43.1%)	300 (37.6%)	371 (48.8%)	
Pure tone average, worse ear (dB HL)	27.3 (16.2)	19.2 (11.4)	35.9 (16.1)	< 0.01
Pure tone average, worse ear degree (dB HL)				< 0.01
Normal (≤ 25)	795 (51.0%)	588 (73.7%)	207 (27.2%)	
Mild (> 25–40)	399 (25.6%)	163 (20.4%)	236 (31.1%)	
Moderate (> 40–60)	310 (19.9%)	45 (5.6%)	265 (34.9%)	
Severe or profound (60+)	54 (3.5%)	2 (0.3%)	52 (6.8%)	
Race				< 0.01
White	1246 (80.0%)	590 (73.9%)	656 (86.3%)	
Minority	312 (20.0%)	208 (26.1%)	104 (13.8%)	
SEP proxy				0.53
Low	363 (23.3%)	177 (22.2%)	186 (24.5%)	
Mid	390 (25.0%)	200 (25.1%	190 (25.0%)	
High	805 (51.7%)	421 (52.8%)	384 (50.5%)	
Marital Status				< 0.01
Married	864 (55.5%)	414 (52.0%)	450 (59.2%)	
Divorced or separated	244 (15.7%)	132 (16.5%)	112 (14.7%)	
Single	233 (15.0%)	157 (19.7%)	76 (10.0%)	
Widowed	217 (13.9%)	95 (11.9%)	122 (16.1%)	
Comorbid conditions (n)				< 0.01
0	310 (19.9%)	198 (24.8%)	112 (14.7%)	
1	790 (50.7%)	400 (50.1%)	390 (51.3%)	
2	377 (24.2%)	172 (21.6%)	205 (27.0%)	
3+	81 (5.2%)	28 (3.5%)	53 (7.0%)	
Noise exposure (+)	768 (49.3%)	339 (42.5%)	429 (56.5%)	< 0.01
Bothersome tinnitus (+)	330 (21.2%)	106 (13.3%)	224 (29.5%)	< 0.01
Diabetes (+)	184 (11.8%)	84 (10.5%)	100 (13.2%)	0.11
Cardiovascular conditions (+)	722 (46.3%)	336 (42.1%)	386 (50.8%)	< 0.01
Smoking				0.06
Never	700 (44.9%)	378 (47.4%)	322 (42.4%)	
Current	186 (11.9%)	99 (12.4%)	87 (11.5%)	
Past	672 (43.1%)	321 (40.2%)	351 (46.2%)	
Body mass index (kg/m2)	27.1 (5.5)	27.0 (5.2)	27.1 (5.5)	0.76
Speech-in-noise scores	5.3 (14.4)	8.4 (11.7)	2.0 (16.1)	< 0.01
SSW (% total error)	4.4 (6.6)	3.5 (5.7)	5.4 (7.3)	< 0.01
More depressive symptoms	11.9 (4.7)	11.3 (4.2)	12.6 (5.1)	< 0.01
More satisfaction in social activities	28.46 (6.3)	29.1 (5.9)	27.7 (6.6)	< 0.01

Note. (+) indicates positive history of condition

### Prevalence of RHHI self-reported hearing difficulty

**Fig. 1:**
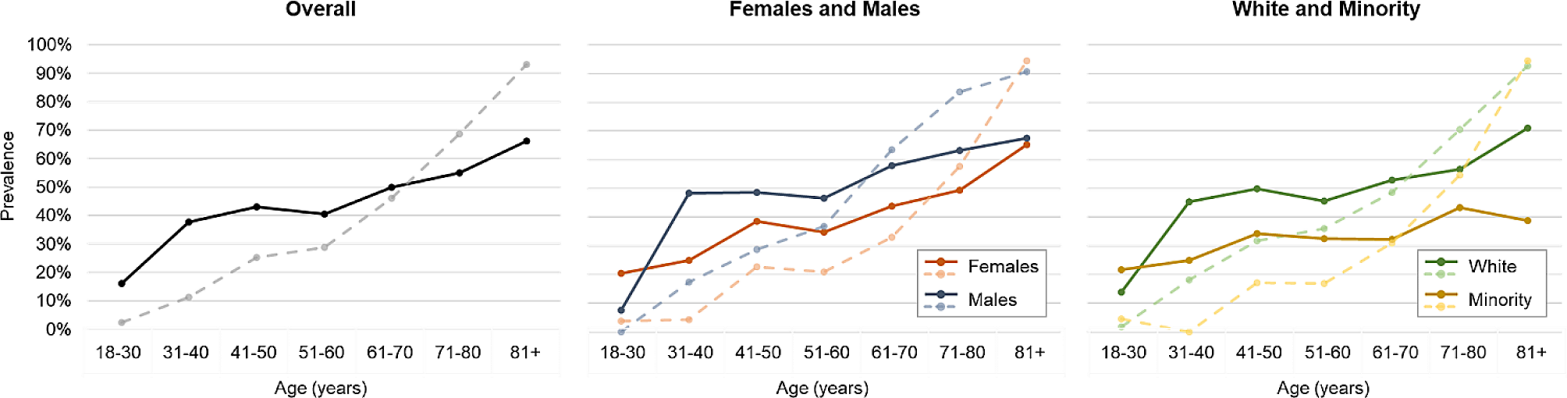
Prevalence estimates of RHHI self-reported hearing difficulty and audiometric hearing loss by age. Descriptive results are presented for the entire sample and stratified by sex and race. Solid lines indicate the prevalence of RHHI self-reported hearing difficulty (score ≥ 6 points) and dashed lines indicate the prevalence of audiometric hearing loss (pure-tone average [PTA] of thresholds at frequencies 0.5, 1.0, 2.0, and 4.0 > 25 dB HL in the worse ear)

Prevalence estimates of RHHI self-reported hearing difficulty and audiometric hearing loss by age, overall and stratified by sex and race, are shown in Fig. 1 and Supplementary Materials 1. In the entire sample, the prevalence of RHHI self-reported hearing difficulty and audiometric hearing loss was 48.8% and 49.0%, respectively, and both increase with age. The following results are descriptive given relatively low sample sizes after stratifying to age group (Supplementary Materials 1). As compared to audiometric hearing loss, younger individuals reported more RHHI self-reported hearing difficulty, and older individuals reported less, and this trend also exists across sex and race categorizations. Across most age groups, males (vs. females) and White (vs. Minority) participants have more audiometric hearing loss and RHHI self-reported hearing difficulty (Fig. 1).

**Fig. 2:**
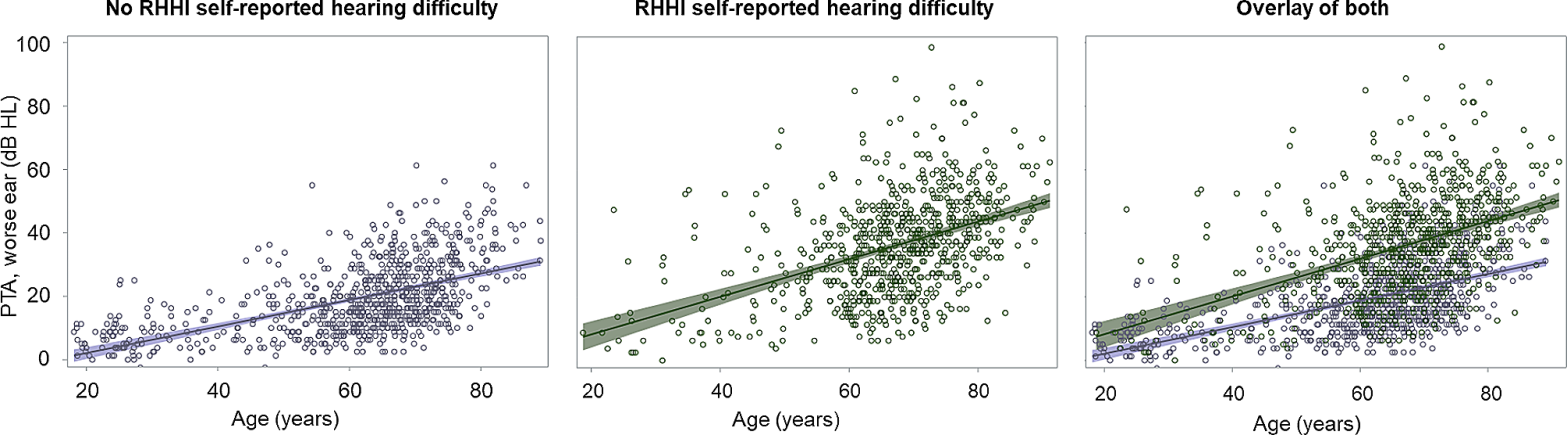
Scatterplot showing age and PTA relationships for participants with and without RHHI self-reported hearing difficulty. Relationships of age and PTA (pure-tone average of thresholds at frequencies 0.5, 1.0, 2.0, and 4.0 > 25 dB HL in the worse ear) are shown for participants without (left panel; purple) and with (middle panel; green) RHHI self-reported hearing difficulty and both overlayed (right panel). Datapoints are fit to a linear regression line, and 95% confidence intervals are shown by shading

Figure 2 shows that on average, PTA increases as age increases. In participants with RHHI self-reported hearing difficulty (Panel 2; regression coefficient: 0.59 [95% CI: 0.51, 0.68]), as compared to those without RHHI self-reported hearing difficulty (Panel 1; regression coefficient: 0.42 [95% CI: 0.38, 0.46]), PTA increases more precipitously with age (*p* < 0.01), and relationships show more variability, particularly in younger and older participants.

### Base models

Table 2 shows results from separate base models adjusted for age, sex, and PTA. In base models, older age, and Minority (vs. White) race were associated with lower odds, whereas higher PTA was associated with higher odds of RHHI self-reported hearing difficulty. More comorbid conditions, positive noise exposure, and bothersome tinnitus were associated with increased odds of RHHI self-reported hearing difficulty. Speech-in-noise scores that are better than predicted, and more satisfaction in social activities were associated with lower odds, whereas more depressive symptoms were associated with higher odds of RHHI self-reported hearing difficulty.

**Table 2:** Factors associated with RHHI self-reported hearing difficulty (score ≥ 6 points) from separate age-sex-PTA adjusted base models

Characteristic	Odds Ratio	95% Confidence Interval		***p***-value
		Lower limit	Upper limit	
Age (per + 1 year)	0.98	0.97	0.99	< 0.01
Female Sex	1.16	0.90	1.48	0.25
PTA worse ear (per + 1 dB)	1.11	1.09	1.12	< 0.01
Minority race	0.63	0.47	0.86	< 0.01
SEP Proxy				
High	REF			
Low	0.93	0.69	1.25	0.63
Mid	0.84	0.63	1.12	0.24
Marital status				
Married	REF			
Divorced/separated	0.95	0.68	1.34	0.78
Single	0.75	0.50	1.12	0.16
Widowed	0.94	0.65	1.37	0.76
Comorbid conditions (n)				
0	REF			
1	1.67	1.20	2.32	< 0.01
2	2.23	1.51	3.28	< 0.01
3+	3.08	1.67	5.68	< 0.01
Noise exposure (+)	1.65	1.26	2.16	< 0.01
Bothersome tinnitus (+)	2.37	1.77	3.19	< 0.01
Diabetes (+)	1.09	0.75	1.57	0.66
Cardiovascular conditions (+)	1.15	0.90	1.47	0.26
Smoking				
Never	REF			
Current	1.17	0.79	1.72	0.43
Past	1.16	0.60	1.50	0.26
Body mass index (kg/m^2^) (per + 1 unit)	1.01	0.99	1.03	0.43
Speech-in-noise scores	0.99	0.98	1.00	0.02
SSW (% total error)	0.99	0.98	1.01	0.55
More depressive symptoms (per + 1 point)	1.05	1.02	1.08	< 0.01
More satisfaction in social activities (per + 1 point)	0.97	0.95	0.98	< 0.01

Note: Age is adjusted for sex and PTA. Sex is adjusted for age and PTA. PTA is adjusted for age and sex. REF = referent group. (+) indicates positive history of condition

### Multivariable model

Table 3 shows results from the multivariable model, which included determinants significant at *p* < 0.10 in base models (described above). In the multivariable model, older age, and Minority (vs. White) race remained associated with lower odds, and female (vs. male) sex and higher PTA were associated with higher odds of RHHI self-reported hearing difficulty. More comorbid conditions, noise exposure, and bothersome tinnitus remained associated with higher odds of RHHI self-reported hearing difficulty. Speech-in-noise scores that were better than predicted were associated with reduced odds, and more depressive symptoms remained associated with higher odds of RHHI self-reported hearing difficulty. Satisfaction in social activities was not associated with RHHI self-reported hearing difficulty after multivariable adjustment (*p* = 0.08).

**Table 3:** Factors associated with RHHI self-reported hearing difficulty (score ≥ 6 points) in a multivariable model

Characteristic	Odds Ratio	95% Confidence Interval		***p***-value
		Lower limit	Upper limit	
Age (per + 1 year)	0.97	0.96	0.98	< 0.01
Female Sex	1.39	1.03	1.86	0.03
PTA worse ear (per + 1 dB)	1.10	1.09	1.12	< 0.01
Minority race	0.68	0.49	0.94	0.02
Comorbid conditions (n)				
0	REF			
1	1.50	1.07	2.11	0.02
2	1.96	1.32	2.93	< 0.01
3+	3.00	1.60	5.62	< 0.01
Noise exposure (+)	1.54	1.16	2.03	< 0.01
Bothersome tinnitus (+)	2.16	1.59	2.93	< 0.01
Speech-in-noise scores	0.99	0.98	1.00	< 0.01
More depressive symptoms (per + 1 point)	1.04	1.01	1.07	0.02
More satisfaction in social activities (per + 1 point)	0.98	0.96	1.00	0.08

Note: Sex forced into multivariable model. REF = referent group. (+) indicates positive history of condition

### Supplementary and sensitivity analyses

Base and multivariable models stratified by sex and race are in Supplementary Materials 2 and 3, respectively. Regarding sex, the factors that differed between the separate multivariable models in females and males were race (significant for females only) and noise exposure (significant for males only). For race, fewer factors, including sex, noise exposure, depressive symptoms, and speech-in-noise, were associated with RHHI self-reported hearing difficulty in Minority (vs. White) participants in multivariable models, although, notably, the sample size for Minority participants was relatively small (*n* = 312). Finally, there were no substantial differences in the presence or the magnitude of associations when the RHHI-S (instead of RHHI) was used as the outcome (results not shown).

## Discussion

This study conducted in a community-based cohort of individuals from across the age range reported the prevalence of RHHI self-reported hearing difficulty to be 48.8% and reported associated factors. In a multivariable model, demographic factors, including older age, female sex and White race, and poorer hearing sensitivity (PTA), more comorbid conditions, noise exposure, bothersome tinnitus, speech-in-noise performance, and more depressive symptoms were associated with self-reported hearing difficulty. This is one of few studies that reports the prevalence and factors associated with self-reported hearing handicap or difficulty, defined by a standardized measure, in the general population [18, 19]. As discussed later, such information could be valuable to inform the interpretation of tools of self-reported hearing difficulty in research and clinical settings.

In this study, factors associated with RHHI self-reported hearing difficulty were rather consistent across the population, though there were some sex- and race-specific differences in associations. For example, noise exposure was associated with RHHI self-reported hearing difficulty in the entire population, and in subsamples of males and White participants, but not in subsamples of females or Minority participants. These differences likely reflect differences in the prevalence of noise exposure in the general population and are consistent with evidence from the literature that prevalence of positive history of noise exposure is highest in White men [41–43]. 

A previous study in this cohort reported the sensitivity and specificity of the RHHI to detect audiometric hearing loss as 72.5% and 74.0%, respectively [12]. Those findings suggest there are other factors that explain relationships between hearing defined by audiometry and the RHHI, and may imply the RHHI captures impacts of hearing loss on functional abilities that are not fully explained by audiometry [6, 44]. In this study, we detected factors associated with RHHI self-reported hearing difficulty, independent of audiometric PTA, as base and multivariable models were adjusted for PTA.

This study shows demographic differences in relationships between audiometric hearing and RHHI self-reported hearing difficulty. Older age was associated with lower odds of RHHI self-reported hearing difficulty, although prevalence of audiometric hearing loss increases with age [1, 14]. Compared to audiometric hearing, younger adults reported more hearing difficulty, whereas older adults reported less hearing difficulty. This is consistent with other studies evaluating relationships of age with audiometric and self-reported hearing on the RHHI or other self-reported questions of hearing [45, 46]. Possible explanations for these findings are that older adults may view hearing loss as a normal part of aging or may attempt to avoid the stigma associated with hearing loss by reporting less hearing difficulty [45, 47]. In terms of sex, females self-report slightly more hearing difficulty (vs. audiometric hearing loss) and this relationship is maintained into older ages, whereas males self-report less hearing difficulty on the RHHI. These sex differences are consistent with previous studies that investigated relationships between audiometry and other self-reported questions of hearing difficulty, including the HHIE [19, 45]. Findings could be explained by sex or gender differences in levels of social engagement, where communication is imperative, which are often higher for females (vs. males) across the adult lifespan [48]. Results from this study suggest that, independent of PTA, individuals of Minority race, most of whom were Black or African American, self-report less hearing difficulty, which could be due to cultural differences in the perceived impacts of hearing loss. Taken together, these differences are consistent with past research suggesting that demographic factors, including age, sex, and race, modify the agreement between self-reported hearing difficulty and audiometric hearing [40]. 

Noise exposure and bothersome tinnitus were associated with increased odds of RHHI self-reported hearing difficulty, independent of PTA. In part, this may be because PTA was calculated from frequencies most important for speech understanding (0.5-4.0 kHz), but not high-frequency hearing (≥ 6 kHz). Tinnitus is associated with high-frequency hearing and often, the first auditory symptoms of noise exposure present as high-frequency hearing loss [49, 50]. Therefore, these associations may, in part, reflect high-frequency hearing thresholds. In this study, we used a speech-frequency PTA to capture hearing loss that is often most impactful to individuals, and to facilitate comparison with other epidemiological studies of hearing loss [1, 14, 15, 51,52]. Speech-in-noise performance that was better than predicted, the measure that was corrected for audibility for frequencies 0.25-8.0 kHz, was also associated with reduced odds of RHHI self-reported hearing difficulty. Speech-in-noise tests capture complex auditory processes and are related to individuals’ perceived hearing [53]. Therefore, the observed relationship with speech-in-noise performance may reflect those complex processes that are not captured by pure-tone audiometry alone.

Physical (more comorbid conditions) and mental health (depressive symptoms) were also associated with RHHI self-reported hearing difficulty. Hearing loss has been associated with poorer physical and mental health in cross-sectional and longitudinal studies [2–4, 54–57]. Possible explanations for this finding are that poor hearing may lead to poorer physical and mental health, or that such conditions co-occur with aging [56, 57]. Importantly, physical and mental health, as well as the RHHI, were measured by self-report, and may be impacted by individuals’ tendencies to report negatively on several measures, often called ‘health pessimism’ [58]. Relationships that may be due to the tendency to report negatively on several measures could apply to other self-reported measures in this study, including tinnitus.

Understanding the prevalence of RHHI self-reported hearing difficulty and associated factors is important for research and clinical care. For example, study results can inform the design and interpretation of epidemiological studies that use the RHHI or RHHI-S, for example, as an outcome measure to determine the impacts of hearing loss on functional abilities in a population, or to measure benefits of hearing loss interventions. Clinically, the RHHI is commonly used as a patient-reported outcome measure, and study results may assist clinicians and researchers in understanding factors associated with RHHI self-reported hearing difficulty. Importantly, there may be situations where individuals choose to use the RHHI instead of the RHHI-S and vice versa. For example, because the RHHI has more questions than the RHHI-S, it could provide additional details relevant to clinical counseling. Conversely, the RHHI-S is shorter, so it could be used when there are strict time limitations in clinical settings or in research, such as in epidemiological studies that collect a wide range of measures [18, 19, 45, 59, 60]. Results from this study suggest that the factors associated with the RHHI and RHHI-S are similar and warrant similar interpretation.

Strengths of this community-based cohort study include its large and diverse sample and comprehensive measures related to hearing and health. This cohort study is similar to other epidemiological studies of ARHL in terms of age and audiometric hearing loss [14, 20, 59]. However, this study, which was conducted using data from participants’ baseline examinations, is limited by its observational and cross-sectional design. Participants in this study were primary White or Black or African American, so it was not possible to evaluate relationships of other races with RHHI self-reported hearing difficulty. Health-related measures were self-reported and thus may be prone to biases. However, it is likely that response bias is consistent across the population, reducing the overall bias in results. There may be other factors related to RHHI self-reported hearing difficulty that were not captured in this study.

## Conclusion

The prevalence of RHHI self-reported hearing difficulty was 48.8%. Factors associated with self-reported hearing difficulty, independent of hearing sensitivity, included demographic and hearing-related factors, physical and mental health. Findings from this study can support and inform the use of the RHHI in research and clinical settings.

### Electronic supplementary material

Below is the link to the electronic supplementary material.

Supplementary Material 1

Supplementary Material 2

Supplementary Material 3

## Data Availability

The datasets generated and/or analyzed during the current study are not publicly available because data collection is ongoing but data are available from the corresponding author on reasonable request.

## Abbreviations

ARHL: Age-related hearing loss
HHIA: Hearing Handicap Inventory for Adults
HHIE: Hearing Handicap Inventory for the Elderly
RHHI: Revised Hearing Handicap Inventory
RHHI-S: Revised Hearing Handicap Inventory-Screening version
PROMIS: Patient-Reported Outcomes Measures Information System
PTA: Pure-tone average
SEP: Socioeconomic position
SPIN: Speech Perception in Noise
SRT: Speech reception threshold
SSW: Staggered spondaic words
