# Supplementary material for: Prevalence of self-reported hearing difficulty on the Revised Hearing Handicap Inventory and associated factors
Source: BMC Geriatr. 2024 Jun 12;24:510. doi: 10.1186/s12877-024-04901-w (PMC11167844; doi:10.1186/s12877-024-04901-w)
Supplement: Supplementary file 1 — Supplementary Material 1 [file 12877_2024_4901_MOESM1_ESM.docx]

**Supplementary Materials 1: Prevalence estimates by demographic factors.**

Supplementary Table 1: Prevalence estimates of RHHI self-reported hearing difficulty and audiometric hearing loss by age group, overall, and by sex and race categorizations.

| **Age group (years)** | **Overall** | | | **Females** | | | **Males** | | | **White** | | | **Minority** | | |
| --- | --- | --- | --- | --- | --- | --- | --- | --- | --- | --- | --- | --- | --- | --- | --- |
|  |  | **Prevalence** | |  | **Prevalence** | |  | **Prevalence** | |  | **Prevalence** | |  | **Prevalence** | |
|  | **n** | **RHHI** | **Audio-metric** | **n** | **RHHI** | **Audio-metric** | **n** | **RHHI** | **Audio-metric** | **n** | **RHHI** | **Audio-metric** | **n** | **RHHI** | **Audio-metric** |
| 18-30 | 81 | 16.0% | 2.5% | 54 | 20.4% | 3.7% | 27 | 7.4% | 0.0% | 58 | 13.8% | 1.7% | 23 | 21.7% | 4.3% |
| 31-40 | 53 | 37.7% | 11.3% | 24 | 25.0% | 4.2% | 29 | 48.3% | 17.2% | 33 | 45.5% | 18.2% | 20 | 25.0% | 0.0% |
| 41-50 | 79 | 43.0% | 25.3% | 44 | 38.6% | 22.7% | 35 | 48.6% | 28.6% | 44 | 50.0% | 31.8% | 35 | 34.3% | 17.1% |
| 51-60 | 202 | 40.6% | 28.7% | 101 | 34.7% | 20.8% | 101 | 46.5% | 36.6% | 125 | 45.6% | 36.0% | 77 | 32.5% | 16.9% |
| 61-70 | 598 | 49.8% | 46.0% | 341 | 43.7% | 32.8% | 257 | 58.0% | 63.4% | 511 | 52.8% | 48.5% | 87 | 32.2% | 31.0% |
| 71-80 | 430 | 55.1% | 68.6% | 251 | 49.4% | 57.8% | 179 | 63.1% | 83.8% | 377 | 56.8% | 70.6% | 53 | 43.4% | 54.7% |
| 81+ | 115 | 66.1% | 93.0% | 72 | 65.3% | 94.4% | 43 | 67.4% | 90.7% | 97 | 71.1% | 92.8% | 18 | 38.9% | 94.4% |
