# Supplementary material for: Prevalence of self-reported hearing difficulty on the Revised Hearing Handicap Inventory and associated factors
Source: BMC Geriatr. 2024 Jun 12;24:510. doi: 10.1186/s12877-024-04901-w (PMC11167844; doi:10.1186/s12877-024-04901-w)
Supplement: Supplementary file 2 — Supplementary Material 2 [file 12877_2024_4901_MOESM2_ESM.docx]

**Supplementary Materials 3: Race-specific models evaluating factors associated with RHHI self-reported hearing difficulty.**

Supplementary Table 6: Factors associated with RHHI self-reported hearing difficulty from separate age-sex-PTA adjusted base models in *White participants* only (n=1246).

|  | **Odds Ratio** | **95% Confidence Interval** | | **p-value** |
| --- | --- | --- | --- | --- |
| **Characteristic** |  | **Lower limit** | **Upper limit** |  |
| Age (per +1 yr) | 0.98 | 0.97 | 0.99 | <0.01 |
| Female Sex | 1.38 | 1.04 | 1.83 | 0.03 |
| PTA worse ear (per +1 dB) | 1.12 | 1.10 | 1.13 | <0.01 |
| SEP Proxy |  |  |  |  |
| High | REF | | | |
| Low | 1.11 | 0.76 | 1.62 | 0.60 |
| Mid | 0.91 | 0.66 | 1.25 | 0.56 |
| Marital status |  |  |  |  |
| Married | REF | | | |
| Divorced/separated | 1.11 | 0.74 | 1.67 | 0.61 |
| Single | 0.68 | 0.41 | 1.14 | 0.14 |
| Widowed | 0.85 | 0.55 | 1.31 | 0.45 |
| Comorbid conditions (n) |  |  |  |  |
| 0 | REF | | | |
| 1 | 1.61 | 1.09 | 2.38 | 0.02 |
| 2 | 1.93 | 1.24 | 3.01 | <0.01 |
| 3+ | 2.48 | 1.28 | 4.81 | <0.01 |
| Noise exposure (+) | 1.73 | 1.26 | 2.37 | <0.01 |
| Bothersome tinnitus (+) | 2.22 | 1.60 | 3.07 | <0.01 |
| Diabetes (+) | 1.05 | 0.65 | 1.69 | 0.85 |
| Cardiovascular conditions (+) | 1.15 | 0.87 | 1.52 | 0.34 |
| Smoking |  |  |  |  |
| Never | REF | | | |
| Current | 1.03 | 0.64 | 1.65 | 0.92 |
| Past | 1.09 | 0.82 | 1.46 | 0.55 |
| Body mass index (kg/m^2^) (per +1 unit) | 1.02 | 0.99 | 1.04 | 0.26 |
| Speech-in-noise scores | 0.99 | 0.98 | 1.00 | <0.01 |
| SSW (% total error) | 1.01 | 0.98 | 1.03 | 0.63 |
| More depressive symptoms (per +1 point) | 1.05 | 1.02 | 1.08 | <0.01 |
| More satisfaction in social activities (per +1 point) | 0.98 | 0.96 | 1.00 | 0.04 |

Note. Age is adjusted for sex and PTA. Sex is adjusted for age and PTA. PTA is adjusted for age and sex. REF=referent group. (+) indicates positive history of condition.

Supplementary Table 7: Factors associated with RHHI self-reported hearing difficulty in a multivariable model in *White participants* only (n=1246).

| **Characteristic** | **Odds Ratio** | **95% Confidence Interval** | | | | **p-value** |
| --- | --- | --- | --- | --- | --- | --- |
|  |  | | **Lower limit** | **Upper limit** |  | |
| Age (per +1 yr) | 0.97 | | 0.96 | 0.99 | <0.01 | |
| Female Sex | 1.65 | | 1.18 | 2.32 | <0.01 | |
| PTA worse ear (per +1 dB) | 1.12 | | 1.09 | 1.13 | <0.01 | |
| Comorbid conditions (n) |  | |  |  |  | |
| 0 | REF | | | | | |
| 1 | 1.51 | | 1.01 | 2.26 | 0.04 | |
| 2 | 1.79 | | 1.14 | 2.83 | 0.01 | |
| 3+ | 2.63 | | 1.34 | 5.17 | <0.01 | |
| Noise exposure (+) | 1.62 | | 1.18 | 2.24 | <0.01 | |
| Bothersome tinnitus (+) | 2.12 | | 1.52 | 2.96 | <0.01 | |
| Speech-in-noise scores | 0.99 | | 0.98 | 1.00 | <0.01 | |
| More depressive symptoms (per +1 point) | 1.04 | | 1.00 | 1.08 | 0.03 | |
| More satisfaction in social activities (per +1 point) | 0.99 | | 0.97 | 1.02 | 0.67 | |

Note. REF=referent group. (+) indicates positive history of condition.

Supplementary Table 8: Factors associated with RHHI self-reported hearing difficulty from separate age-sex-PTA adjusted base models in *Minority participants* only (n=312).

| **Characteristic** | **Odds Ratio** | **95% Confidence Interval** | | **p-value** |
| --- | --- | --- | --- | --- |
|  |  | **Lower limit** | **Upper**  **limit** |  |
| Age (per +1 yr) | 0.98 | 0.96 | 1.00 | 0.08 |
| Female Sex | 0.75 | 0.44 | 1.27 | 0.28 |
| PTA worse ear (per +1 dB) | 1.07 | 1.04 | 1.09 | <0.01 |
| SEP Proxy |  |  |  |  |
| High | REF | | | |
| Low | 0.87 | 0.47 | 1.61 | 0.67 |
| Mid | 0.70 | 0.34 | 1.46 | 0.35 |
| Marital status |  |  |  |  |
| Married | REF | | | |
| Divorced/separated | 0.85 | 0.41 | 1.75 | 0.65 |
| Single | 0.99 | 0.47 | 2.07 | 0.98 |
| Widowed | 1.95 | 0.89 | 4.39 | 0.11 |
| Comorbid conditions (n) |  |  |  |  |
| 0 | REF | | | |
| 1 | 1.79 | 0.95 | 3.37 | 0.07 |
| 2+ | 3.74 | 1.64 | 8.60 | <0.01 |
| Noise exposure (+) | 1.15 | 0.88 | 2.71 | 0.13 |
| Bothersome tinnitus (+) | 2.98 | 1.36 | 6.50 | <0.01 |
| Diabetes (+) | 1.81 | 0.98 | 3.34 | 0.06 |
| Cardiovascular conditions (+) | 1.85 | 1.03 | 3.32 | 0.04 |
| Smoking |  |  |  |  |
| Never | REF | | | |
| Current | 1.75 | 0.85 | 3.58 | 0.13 |
| Past | 1.43 | 0.78 | 2.62 | 0.25 |
| Body mass index (kg/m^2^) (per +1 unit) | 1.03 | 0.99 | 1.07 | 0.20 |
| Speech-in-noise scores | 1.00 | 0.98 | 1.03 | 0.72 |
| SSW (% total error) | 0.99 | 0.95 | 1.03 | 0.54 |
| More depressive symptoms (per +1 point) | 1.07 | 1.04 | 1.09 | <0.01 |
| More satisfaction in social activities (per +1 point) | 0.94 | 0.90 | 0.97 | <0.01 |

Note. Age is adjusted for sex and PTA. Sex is adjusted for age and PTA. PTA is adjusted for age and sex. REF=referent group. (+) indicates positive history of condition. Number of comorbid conditions categories were collapsed to 0, 1, 2+ given limited sample size in analyses.

Supplementary Table 9: Factors associated with RHHI self-reported hearing difficulty in a multivariable model in *Minority participants* only (n=312).

| **Characteristic** | **Odds Ratio** | **95% Confidence Interval** | | **p-value** |
| --- | --- | --- | --- | --- |
|  |  | **Lower limit** | **Upper limit** |  |
| Age (per +1 yr) | 0.96 | 0.94 | 0.99 | <0.01 |
| Female Sex | 0.74 | 0.41 | 1.32 | 0.31 |
| PTA worse ear (per +1 dB) | 1.07 | 1.05 | 1.10 | <0.01 |
| Comorbid conditions (n) |  |  |  |  |
| 0 | REF | | | |
| 1 | 1.56 | 0.80 | 3.05 | 0.20 |
| 2+ | 2.75 | 1.14 | 6.65 | 0.02 |
| Bothersome tinnitus (+) | 2.71 | 1.19 | 6.16 | 0.02 |
| Cardiovascular conditions (+) | 1.49 | 0.79 | 2.80 | 0.22 |
| Diabetes (+) | 1.47 | 0.76 | 2.85 | 0.26 |
| More depressive symptoms (per +1 point) | 1.04 | 0.98 | 1.10 | 0.26 |
| More satisfaction in social activities (per +1 point) | 0.96 | 0.91 | 1.00 | 0.07 |

Note. REF=referent group. (+) indicates positive history of condition. Number of comorbid conditions categories were collapsed to 0, 1, 2+ given limited sample size in analyses.
