# Supplementary material for: Prevalence of self-reported hearing difficulty on the Revised Hearing Handicap Inventory and associated factors
Source: BMC Geriatr. 2024 Jun 12;24:510. doi: 10.1186/s12877-024-04901-w (PMC11167844; doi:10.1186/s12877-024-04901-w)
Supplement: Supplementary file 3 — Supplementary Material 3 [file 12877_2024_4901_MOESM3_ESM.docx]

**Supplementary Materials 2: Sex-specific models evaluating factors associated with RHHI self-reported hearing difficulty.**

Supplementary Table 2: Factors associated with RHHI self-reported hearing difficulty from separate age-sex-PTA adjusted base models in *females* only (n=887).

| **Characteristic** | **Odds Ratio** | **95% Confidence Interval** | | | | **p-value** |
| --- | --- | --- | --- | --- | --- | --- |
|  |  | | **Lower limit** | **Upper limit** |  | |
| Age (per +1 yr) | 0.98 | | 0.97 | 1.00 | <0.01 | |
| PTA worse ear (per +1 dB) | 1.10 | | 1.09 | 1.12 | <0.01 | |
| Minority race | 0.53 | | 0.36 | 0.78 | <0.01 | |
| SEP Proxy |  | |  |  |  | |
| High | REF | | | | | |
| Low | 0.91 | | 0.61 | 1.35 | 0.64 | |
| Mid | 0.78 | | 0.53 | 1.13 | 0.19 | |
| Marital status |  | |  |  |  | |
| Married | REF | | | | | |
| Divorced/separated | 0.96 | | 0.62 | 1.47 | 0.84 | |
| Single | 0.66 | | 0.40 | 1.09 | 0.11 | |
| Widowed | 0.87 | | 0.57 | 1.33 | 0.52 | |
| Comorbid conditions (n) |  | |  |  |  | |
| 0 | REF | | | | | |
| 1 | 1.43 | | 0.89 | 2.29 | 0.14 | |
| 2 | 2.02 | | 1.20 | 3.39 | <0.01 | |
| 3+ | 3.03 | | 1.45 | 6.35 | <0.01 | |
| Noise exposure (+) | 1.34 | | 0.95 | 1.89 | 0.09 | |
| Bothersome tinnitus (+) | 2.53 | | 1.70 | 3.78 | <0.01 | |
| Diabetes (+) | 0.93 | | 0.58 | 1.48 | 0.75 | |
| Cardiovascular conditions (+) | 1.00 | | 0.72 | 1.38 | 0.99 | |
| Smoking |  | |  |  |  | |
| Never | REF | | | | | |
| Current | 1.82 | | 0.45 | 1.51 | 0.53 | |
| Past | 0.99 | | 0.71 | 1.37 | 0.96 | |
| Body mass index (kg/m^2^) (per +1 unit) | 1.01 | | 0.98 | 1.03 | 0.58 | |
| Speech-in-noise scores | 0.99 | | 0.91 | 1.00 | 0.02 | |
| SSW (% total error) | 1.00 | | 0.97 | 1.02 | 0.77 | |
| More depressive symptoms (per +1 point) | 1.05 | | 1.02 | 1.089 | <0.01 | |
| More satisfaction in social activities (per +1 point) | 0.96 | | 0.94 | 0.99 | <0.01 | |

Note. Age is adjusted for PTA. PTA is adjusted for age. REF=referent group. (+) indicates positive history of condition.

Supplementary Table 3: Factors associated with RHHI self-reported hearing difficulty in a multivariable model in *females* only (n=887).

| **Characteristic** | **Odds Ratio** | **95% Confidence Interval** | | **p-value** |
| --- | --- | --- | --- | --- |
|  |  | **Lower limit** | **Upper**  **limit** |  |
| Age (per +1 yr) | 0.97 | 0.96 | 0.99 | <0.01 |
| PTA worse ear (per +1 dB) | 1.10 | 1.08 | 1.12 | <0.01 |
| Minority race | 0.57 | 0.38 | 0.85 | <0.01 |
| Comorbid conditions (n) |  |  |  |  |
| 0 | REF | | | |
| 1 | 1.44 | 0.89 | 2.35 | 0.14 |
| 2 | 1.90 | 1.10 | 3.26 | 0.02 |
| 3+ | 3.19 | 1.15 | 6.83 | <0.01 |
| Noise exposure (+) | 1.28 | 0.89 | 1.84 | 0.18 |
| Bothersome tinnitus (+) | 2.32 | 1.53 | 3.53 | <0.01 |
| Speech-in-noise scores | 0.98 | 0.97 | 0.99 | <0.01 |
| More depressive symptoms (per +1 point) | 1.04 | 1.00 | 1.00 | 0.05 |
| More satisfaction in social activities (per +1 point) | 0.98 | 0.95 | 1.01 | 0.17 |

Note. REF=referent group. (+) indicates positive history of condition.

Supplementary Table 4: Factors associated with RHHI self-reported hearing difficulty from separate age-sex-PTA adjusted base models in *males* only (n=671).

| **Characteristic** | **Odds Ratio** | **95% Confidence Interval** | | **p-value** |
| --- | --- | --- | --- | --- |
|  |  | **Lower limit** | **Upper**  **limit** |  |
| Age (per +1 yr) | 0.97 | 0.96 | 0.99 | <0.01 |
| PTA worse ear (per +1 dB) | 1.11 | 1.09 | 1.13 | <0.01 |
| Minority race | 0.86 | 0.52 | 1.43 | 0.56 |
| SEP Proxy |  |  |  |  |
| High | REF | | | |
| Low | 0.93 | 0.59 | 1.47 | 0.75 |
| Mid | 0.90 | 0.57 | 1.44 | 0.67 |
| Marital status |  |  |  |  |
| Married | REF | | | |
| Divorced/separated | 0.90 | 0.51 | 1.59 | 0.71 |
| Single | 0.92 | 0.46 | 1.83 | 0.81 |
| Widowed | 1.19 | 0.48 | 2.97 | 0.70 |
| Comorbid conditions (n) |  |  |  |  |
| 0 | REF | | | |
| 1 | 1.93 | 1.22 | 30.6 | <0.01 |
| 2 | 2.38 | 1.31 | 4.32 | <0.01 |
| 3+ | 2.37 | 0.73 | 7.67 | 0.15 |
| Noise exposure (+) | 2.32 | 1.48 | 3.62 | <0.01 |
| Bothersome tinnitus (+) | 2.20 | 1.41 | 3.42 | <0.01 |
| Diabetes (+) | 1.42 | 0.77 | 2.59 | 0.26 |
| Cardiovascular conditions (+) | 1.38 | 0.95 | 2.01 | 0.09 |
| Smoking |  |  |  |  |
| Never | REF | | | |
| Current | 1.61 | 0.94 | 2.75 | 0.08 |
| Past | 1.53 | 1.01 | 2.31 | 0.05 |
| Body mass index (kg/m^2^) (per +1 unit) | 1.01 | 0.97 | 1.05 | 0.55 |
| Speech-in-noise scores | 0.99 | 0.98 | 1.01 | 0.31 |
| SSW (% total error) | 0.99 | 0.97 | 1.02 | 0.58 |
| More depressive symptoms (per +1 point) | 1.05 | 1.01 | 1.09 | 0.02 |
| More satisfaction in social activities (per +1 point) | 0.97 | 0.94 | 1.00 | 0.02 |

Note. Age is adjusted for PTA. PTA is adjusted for age. REF=referent group. (+) indicates positive history of condition.

Supplementary Table 5: Factors associated with RHHI self-reported hearing difficulty in a multivariable model in *males* only (n=671).

| **Characteristic** | **Odds Ratio** | **95% Confidence Interval** | | **p-value** |
| --- | --- | --- | --- | --- |
|  |  | **Lower limit** | **Upper**  **limit** |  |
| Age (per +1 yr) | 0.97 | 0.95 | 0.99 | <0.01 |
| PTA worse ear (per +1 dB) | 1.11 | 1.09 | 1.13 | <0.01 |
| Comorbid conditions (n) |  |  |  |  |
| 0 | REF | | | |
| 1 | 1.61 | 1.00 | 2.61 | 0.05 |
| 2 | 2.06 | 1.11 | 3.84 | 0.02 |
| 3+ | 2.06 | 0.63 | 6.79 | 0.23 |
| Noise exposure (+) | 2.05 | 1.30 | 3.25 | <0.01 |
| Bothersome tinnitus (+) | 2.10 | 1.33 | 3.34 | <0.01 |
| Cardiovascular conditions (+) | 1.30 | 0.88 | 1.93 | 0.19 |
| Smoking |  |  |  |  |
| Never | REF | | | |
| Current | 1.65 | 0.93 | 2.91 | 0.09 |
| Past | 1.38 | 0.89 | 2.13 | 0.15 |
| More depressive symptoms (per +1 point) | 1.03 | 0.98 | 1.08 | 0.21 |
| More satisfaction in social activities (per +1 point) | 0.99 | 0.95 | 1.02 | 0.39 |

Note. REF=referent group. (+) indicates positive history of condition.
